# Children with Genetically Confirmed Hereditary Spastic Paraplegia: A Single-Center Experience

**DOI:** 10.3390/children12101332

**Published:** 2025-10-04

**Authors:** Seyda Besen, Yasemin Özkale, Murat Özkale, Sevcan Tuğ Bozdoğan, Özlem Alkan, Serdar Ceylaner, İlknur Erol

**Affiliations:** 1 Department of Pediatric Neurolog, Adana Dr. Turgut Noyan Teaching and Medical Research Center, Baskent University Faculty of Medicine, 01120 Adana, Turkey; seydabesen@baskent.edu.tr (S.B.); ilknur_erol@yahoo.com (İ.E.); 2 Department of Pediatrics, Adana Dr. Turgut Noyan Teaching and Medical Research Center, Baskent University Faculty of Medicine, Baraj Yolu 1 Durak, 01120 Seyhan, Turkey; 3 Department of Pediatric Intensive Care, Adana Dr. Turgut Noyan Teaching and Medical Research Center, Baskent University Faculty of Medicine, 01120 Adana, Turkey; muratozkale@baskent.edu.tr; 4 Department of Medical Genetics, Faculty of Medicine, Çukurova University, 01330 Adana, Turkey; stbozdogan@cu.edu.tr; 5 Department of Radiology, Adana Dr. Turgut Noyan Teaching and Medical Research Center, Baskent University Faculty of Medicine, 01120 Adana, Turkey; alkano@baskent.edu.tr; 6 Department of Medical Genetics, Faculty of Medicine, Lokman Hekim University, 06510 Ankara, Turkey; serdar.ceylaner@lokmanhekim.edu.tr

**Keywords:** hereditary spastic paraplegia, child, *KIDINS220*, *CYP7B1*, *TFG*, *GBA2*, *SPG11*

## Abstract

**Highlights:**

**What are the main findings?**

- We identified a new inheritance pattern, a novel variants and a different clinical phenotype in some forms of hereditary spastic paraplegia.

**What is the implication of the main finding?**

- The new inheritance patterns, new variants and phenotypes identified will contribute to the clinical and genetic diagnosis and monitoring of HSP.

**Abstract:**

**Objective:** The classification of hereditary spastic paraplegia (HSP) is based on genetics, and the number of genetic loci continues to increase with new genetic descriptions. Additionally, the number of new variants in known mutations continues to increase. In this paper, we aim to report our experience with genetically confirmed HSPs. **Methods:** We retrospectively evaluated 10 consecutive children with genetically confirmed HSPs. **Results:** In this study, we identified six novel mutations, including spastic paraplegia 11 (*SPG11*), glucosylceramidase beta 2 (*GBA2)*, chromosome 19 open reading frame 12 (*C19orf12*), 1 in each of the Cytochrome P450 family 7 subfamily B member 1 (*CYP7B1*) genes, and two different mutations in the intropomyosin-receptor kinase fused gene (*TFG*) gene. We also identified different clinical phenotypes associated with known mutations. **Conclusions:** Heterozygous mutations with *GBA2* and *SPG11* mutation-related HSP are reported for the first time, expanding the known inheritance patterns. We report a novel homozygous chromosome 19 open reading frame 12 (*C19orf12*) mutation resulting in iron accumulation in the brain, broadening the genetic variants and clinical findings. We determine the first Turkish patients with carnitine palmitoyltransferase IC (*CPT1C*) and *TFG* gene mutation-related pure HSP. A pure form of HSP with two novel TFG gene mutations is also identified for the first time. We report the first Turkish patient with kinase D-interacting substrate of 220 kDa (*KIDINS220*) gene, broadening the clinical spectrum of *KIDINS220* variant-related disorders to encompass certain HSPs. Moreover, a novel variant in the oxysterol7-hydroxylase (*CYP7B1*) gene is reported, expanding the genetic variants and clinical findings relating to *SPG5*.

## 1. Introduction

Hereditary spastic paraplegias (HSPs) refer to a group of rare inherited diseases characterized by progressive degeneration of the corticospinal tracts and presenting with spasticity and weakness of the lower extremities. The prevalence of the disease is 0.1–9.6 per 100,000 people. The clinical manifestation generally starts in childhood and progresses slowly into adulthood, resulting in disability. Clinically, HSP with only bladder involvement is classified as the “pure” form, while that with additional neurological or systemic abnormalities other than bladder involvement is classified as the “complicated” form [1,2,3]. At present, the classification of HSPs is increasingly based on genetics, especially given the phenotypic heterogeneity of HSP within members of the same family harboring the same genetic defect. HSPs are genetically classified according to the mode of inheritance, chromosomal locus, and causative mutation. The inheritance patterns are autosomal dominant (AD), autosomal recessive (AR), and X-linked dominant or mitochondrial inheritance. With the progression of molecular diagnostic techniques, more than 90 causative mutations have been identified, and this number is increasing. They are named *SPG*s based on their genetic loci, numbered as *SPG1*, *SPG2*, *SPG3*, and so on, with the numbering of *SPG*s based on the order of locus discovery and not on the mechanism of genetic transmission. The correlation of clinical classification (pure or complex) with genetic classification (SPG type) is not possible, as some genetic HSPs are associated with both pure and complex phenotypes; moreover, a specific form of HSP may be caused by both dominant and recessive variants in the same gene [1,2,3]. In addition, the distribution and frequency of genetic mutations in HSPs varies from region to region and shows high clinical and genetic heterogeneity. This study provides a new perspective for the diagnosis and genetic distribution of HSPs in Turkey. As intensive studies on targeted therapies and even gene therapies are ongoing for some forms of HSP, the identification of genetic loci is crucial for the design of targeted therapies [1,2,3,4,5,6].

## 2. Materials and Methods

We retrospectively evaluated 10 consecutive children with genetically confirmed HSP at the pediatric neurology division of Baskent University, Adana Hospital, between February 2019 and April 2023. We collected demographic data and details on neurologic status, electroencephalography (EEG), electroneuromyography (ENMG), magnetic resonance imaging (MRI) of the brain and spinal cord, and all laboratory, metabolic screening, genetic analysis, and treatment results from the medical records of each patient.

DNA Sequencing

DNA isolation was performed using an automated magnetic separator. Exome enrichment was performed using the Twist Comprehensive Human Exome kit (San Francisco, California), according to the manufacturer’s instructions. The prepared library was sequenced on MGI-T7 at 80–100× on-target depth with 150 bp paired-end sequencing at the Intergen Genetic Diagnostic Centre in Ankara, Turkey. Bioinformatics analyses were performed using an in-house developed workflow derived from GATK best practices at Intergen Genetic Diagnostic Centre (Ankara, Turkey). MGI adapter sequences were cleaned from raw reads using cutadapt [7]. Alignment to (GRCh38) was performed using BWA-MEM 0.7.17 [8]. Subsequent sorting, duplicate marking, and base score recalibration steps were performed using GATK. Variant calling was performed using GATK HaplotypeCaller (Variant calling software, Broad Institute of MIT and Harvard, Cambridge, MA, USA) and low-quality variants were eliminated based on strand bias, read depth, and call quality parameters, as well as other related parameters. Copy number variations were inferred using GATK GermlineCNVCaller, Institute of MIT and Harvard, Cambridge, MA, USA [9].

Variant Analysis and Classification

High-quality variants were subjected to functional annotation using Variant Effect Predictor from ENSEMBL, Cambridge, UK [10]. Rare variants (MAF < 1%) with high impact, unknown significance, and/or potential splice effects were prioritized. Other variants with potential effects on the observed phenotype were also analyzed. Variants of interest were visually checked on IGV [11] and compared against an in-house disease variant database by Intergen Genetic Diagnostic Centre (Ankara, Turkey). Potential candidates were confirmed using targeted sequencing on the Illumina MiSeq platform, San Diego, California, USA at Intergen Genetic Diagnostic Centre (Ankara, Turkey). Confirmed candidate variants were also tested with the same methods amongst members of each patient’s family.

Genetic studies were performed for all cases to investigate the genetic cause of HSP. In cases where both the inheritance pattern and the pathogenicity of the variant were demonstrated, the diagnosis was quickly confirmed through familial screening. However, in cases where the inheritance pattern did not match or pathogenicity could not be clearly demonstrated, after excluding all metabolic and genetic causes that could cause HSP through extended familial screening, and after MRI images were found to be consistent, the variant was considered the possible cause of HSP.

Our study was approved by the Institutional Review Board and Ethics Committee of Baskent University, with approval number KA23/165. Written informed consent was obtained from the parents of all participants.

## 3. Results

**Figure 1:**
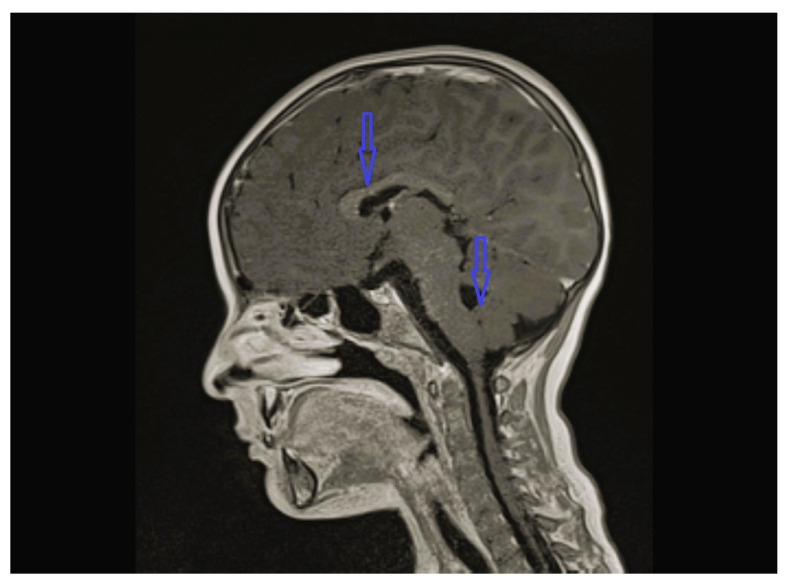
Brain MRI of case 1; the findings were consistent with hypoplasia of the vermis, cerebellum, and corpus callosum.

**Figure 2:**
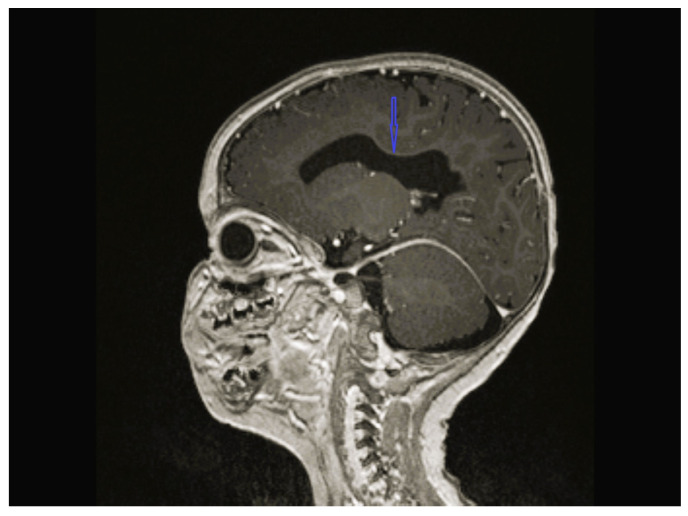
Brain MRI of case 2 revealing hypoplasia of the corpus callosum.

**Figure 3:**
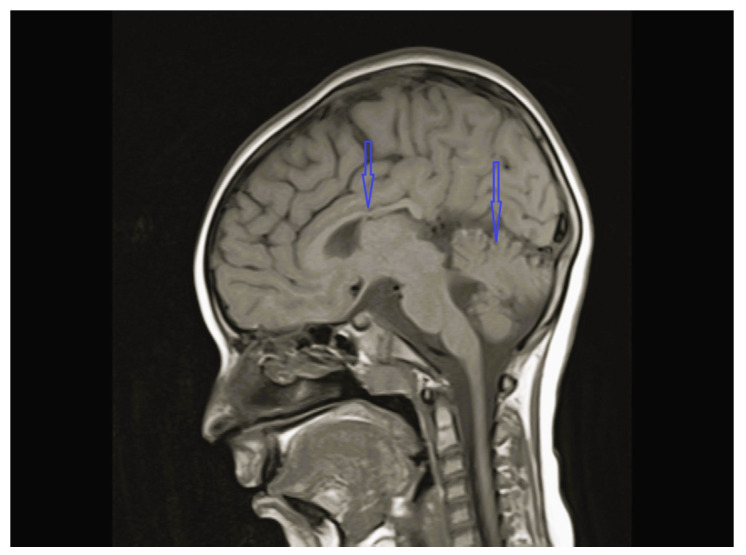
Brain MRI of case 3 revealing cerebellar and corpus callosum hypoplasia.

**Figure 4:**
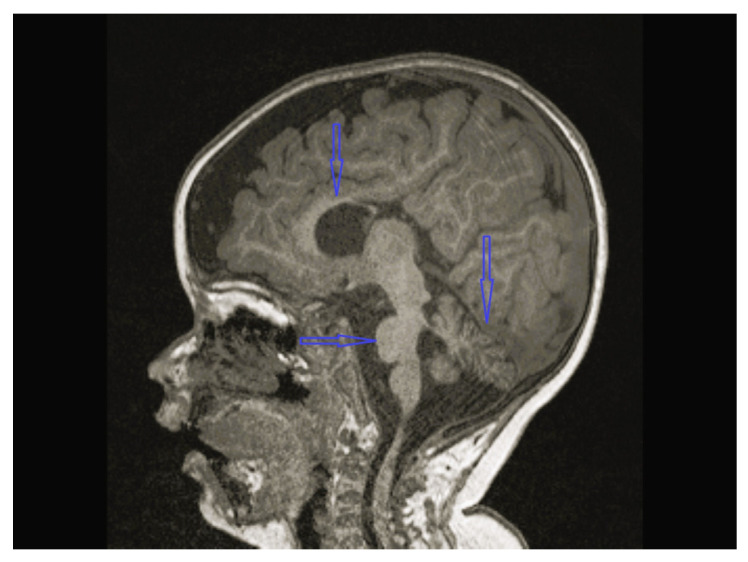
Brain MRI of case 8 revealing vermian, corpus callosum and brainstem hypoplasia.

**Figure 5:**
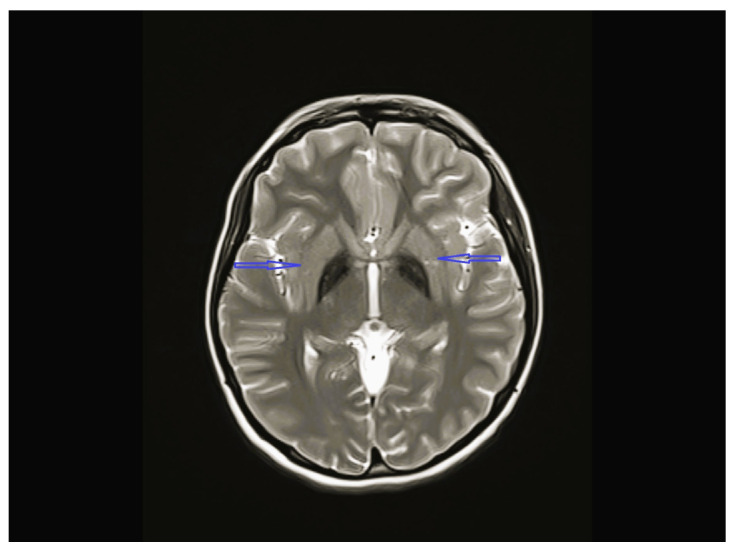
Brain MRI of case 4 revealing bilateral globus pallidus, T2 hypointensity and iron accumulation.

The age of onset ranged from the time of birth to 12 years 1 month; five patients were less than 2 years old at the time of onset. The follow-up period of the patients was 8 months to 12 years 9 months. Except for one, all patients were admitted with lower extremity spasticity, weakness, brisk reflexes, and motor delay. Additionally, seizure, ataxia, speech delay, failure to thrive, congenital hip dislocation, and intellectual disability were observed in one patient each. Three patients had a neurogenic bladder, five of the patients had brain imagining abnormalities, and the MRI of four patients was characterized by corpus callosum hypoplasia and/or accompanying vermian, cerebellar, or brainstem hypoplasia (Figure 1, Figure 2, Figure 3 and Figure 4, cases 1, 2, 3, 8). One patient had bilateral globus pallidus and substantia nigra T2 hypointensity with iron accumulation (Figure 5, case 4). Six of the patients were considered to have pure HSP (Table 1). Genetic analyses revealed pathogenic mutations in the SPG genes. Five patients had six novel mutations, including SPG11 NM_001160227.2 c.6730C > T heterozygote p.L2244F, GBA2 NM_020944.3 c.1688-2A > C, C19orf12 NM_001031726.3 c.385C > T Homozygote p.Q129 *, TFG NM_001195478.1 c.269-8_269-4dup homozygote, TFG NM_001195478.1 c.288_297 delCCTTGAATCAinsTGACTTG homozygote, and CYP7B1 NM_004820.5 c.1346G > A Homozygote p.C449y (HSP43). The demographic, clinical, neuroimaging, laboratory, metabolic screening, EEG, ENMG, genetic, and treatment data of the patients are detailed in Table 1 and Table 2. Since the study was retrospective and patient ages varied, similar developmental assessment and spasticity scale results could not be obtained in all patients.

## 4. Discussion

The classification of HSP is based on genetics, especially given the phenotypic heterogeneity of HSP within the same family with the same genetic defect. Some forms of HSP are associated with both pure and complex phenotypes. The number of genetic loci continues to increase with new genetic descriptions, and the number of new variants in known mutations continues to increase. In many HSP types, both monoallelic and biallelic mutations of the genes have been shown to cause disease. However, this study demonstrates that monoallelic mutations in *GBA2* and *SPG1* cause possible dominantly inherited forms of HSP for the first time in the literature. In this study, we retrospectively evaluated 10 consecutive children with genetically confirmed HSP and identified six novel mutations and also different clinical phenotypes of known and novel mutations. In addition, possible targeted treatments have been initiated for our patients diagnosed with *SPG83* and *SPG5* (Table 1).

*SPG11* mutation-related HSP is one of the most common types of AR-complicated HSP. *SPG11* is caused by mutations in the *SPG11* gene, which encodes a potential vesicle traffic-associated transmembrane protein, spataxin. Accompanying findings include cognitive impairment, thin corpus callosum, white matter abnormalities, dysarthria, nystagmus, and upper extremity involvement [9,10,11]. We detected a novel *SPG11 c.6730C > T heterozygous (p.L2244F)* mutation in case 1. All reported cases with *SPG11* gene mutations related to HSP are seen as homozygous or compound heterozygous mutations [12,13,14]. Case 1 possibly had pure HSP associated with heterozygous *SPG11*, as no other cause could be ascertained from the patient’s spinal cord MRI, metabolic scans, and whole-exome sequencing (WES) analysis other than brain MRI, which was consistent with hypoplasia of the vermis, cerebellum, and corpus callosum (Figure 1) (Table 2). Although the mother also had the same variant in heterozygous state, their father, sisters, and mother’s siblings were normal. An unclear genetic situation remains, such as the incomplete penetrance or variable expression patterns seen in other AD disorders. As we could not perform functional analyses, we could not determine the pathogenicity of the variant clearly. However, as no other cause could be shown, the case was accepted as the first possible heterozygous mutated *SPG11* case.

*SPG46*—one of the AR-complicated HSPs—is clinically characterized by spasticity and weakness of the lower limbs, intellectual disability, congenital bilateral cataract, thin corpus callosum, and hypogonadism in males [15,16]. Case 2 had a novel de novo heterozygous in silico pathogenic variant *GBA2 NM_020944.3 c.1688-2A > C*. His clinical symptoms included axial hypotonia, intellectual disability, spasticity, and hyperreflexia in the lower limbs. He was accepted as AD-inherited *GBA2*-associated HSP, as no other cause could be found in the patient’s spinal cord MRI, metabolic scans, and WES analysis other than brain MRI, which revealed hypoplasia of the corpus callosum (Figure 2) (Table 2). Therefore, a novel heterozygous mutation with possible *GBA2*-associated HSP was reported for the first time.

Heterozygous or homozygous variants in Kinesin Family Member 1A (*KIF1A*) underlie a wide spectrum of neurodevelopmental and neurodegenerative disorders, ranging from pure to complex forms of HSP, as well as ataxic phenotype and other “atypical” phenotypes in a low proportion of patients. Thus, the term “*KIF1A*-related disorder” may be generally used [15,17,18]. *SPG30* has been associated with both dominant and recessive mutations of the *KIF1A* gene. The age of onset in both AR and AD *SPG30* is highly variable from congenital to adult onset [17,18]. The main hypothesis explaining the observed phenotypic differences speculates that a mild alteration in axonal transport affects only the longest axons, causing a mild HSP phenotype, whereas a greater impairment may be associated with a more severe disorder involving different neuronal cells [17,18]. Case 3 had a *KIF1A* mutation related to pure HSP with cerebellar and corpus callosum hypoplasia on brain MRI (Figure 3) (Table 2).

*C19orf12* encodes a mitochondrial small transmembrane protein that causes a spectrum of related conditions, including mitochondrial membrane protein-associated neurodegeneration (MPAN), *AR-SPG43*, and amyotrophic lateral sclerosis (ALS)-like phenotypes [19,20]. MPAN is characterized by spasticity, Parkinsonism unresponsive to L-DOPA therapy, psychiatric features, optic atrophy, cognitive decline, and brain iron deposits. *SPG43* involves spastic paraplegia with lower motor neuron features without vision loss and brain iron accumulation, or with vision loss and evidence of brain iron accumulation but without extrapyramidal features (dystonia and Parkinsonism) [20,21,22]. Only four studies on *SPG43* have been reported in the literature. Case 4 was a 16-year-old girl who presented with intellectual disability, spastic paraparesis, hyperreflexia, ataxia, pes cavus, and hammer toe deformity in both feet. Brain MRI showed T2 signal loss, which might be secondary to iron deposition in the bilateral globus pallidus and substantia nigra (Figure 5) (Table 2). Additionally, a novel C19orf12 c.385C > T (p.Q1239 *) (p.Gln129Ter) homozygous in silico pathogenic mutation was detected using WES. This patient was diagnosed with SPG43 due to the lack of extrapyramidal features (Table 1).

To date, *SPG73* has only been identified in two families with variations in the neuronal isoform of the CPT1C [23,24]. *SPG73* was first reported as a pure form of AD-HSP characterized by adult-onset slow progression in an Italian family in 2015 [23]. However, Hong et al. have reported a Chinese family with relatively benign clinical course and congenital onset [24]. Case 5 is the first Turkish patient with *CPT1C* mutation related to pure HSP in the literature (Table 1 and Table 2), and is also the youngest patient diagnosed with *SPG73*. His father had the same mutation, without any neurological symptoms. The mother of the Chinese patients reported by Hong et al. also had the *CPT1C* mutation but presented only hyperreflexia and mild extensor plantar response, without any other symptoms.

*TFG* has been linked to diverse hereditary neurodegenerative disorders, including AR-inherited *SPG57* and hereditary motor and sensory neuropathy, Okinawa type. *SPG57* is a complicated form of spastic paraplegia, characterized by slow, gradual, and progressive weakness; spasticity of the lower limbs; and neurologic findings including seizures, dementia, amyotrophy, extrapyramidal disturbance, cerebral or cerebellar atrophy, optic atrophy, and peripheral neuropathy, as well as extra neurological manifestations. The rate of progression and the severity of symptoms are quite variable [25,26]. The protein product of the TFG protein comprises three known domains, including a Phox-Bem1p (PB1) domain, a coiled-coil domain, and a proline- and glutamine-rich domain. The clinical variation in *SPG57* is associated with different mutations in these three domains of the *TFG* gene. To date, nine families with *SPG57* and five pathogenic variants in the *TFG* gene have been reported. Case 6 had two different homozygous mutations in the *TFG* gene, which are novel mutations; furthermore, the patient’s parents were also heterozygous for these two mutations (Table 2). This case is the first Turkish patient with a *TFG* gene mutation reported in the literature. Although all cases described to date had complex HSP, this is also the first case having pure HSP associated with *TFG* in the literature. This is also the first case having pure HSP associated with homozygous *TFG* in the literature. However, new neurological or extraneurological findings might be reported in the following years, as the patient was only 6 years of age. Xu Ling et al. also reported a patient with a novel heterozygous *TFG variant (NM_006070.6: c.125G > A (p.R42Q))* with pure HSP. They suggest that autophagy impairment may serve as a common pathomechanism among different clinical phenotypes caused by TFG mutations [27].

Although *SPG4* is the most common form of AD-related pure form of HSP, complex forms are also seen, though rarely. SPG4 is associated with the spastin (SPAST) gene, which encodes a member of the ATPases associated with a variety of cellular activities (AAA) protein family. This protein family shares an ATPase domain and has roles in diverse cellular processes, including membrane trafficking, intracellular motility, organelle biogenesis, protein folding, and proteolysis. Although *SPG4* usually has adult onset, the age of onset extends from the time of birth to the eighth decade due to incomplete penetrance. Most of the de novo SPAST variants cause early-onset severe, complex *SPG4* [28,29]. Case 7 had a very rare form of *SPG4* related to a familial SPAST mutation. It was a pure form, and the time of onset of clinical manifestation was infancy.

*KIDINS220* encodes a conserved scaffold protein that controls axonal and dendritic maturation [30]. Variants in the *KIDINS220* gene cause a spectrum of disorders such as AD spastic paraplegia, intellectual disability, nystagmus, and obesity (SINO) syndrome; AR ventriculomegaly; and arthrogryposis [31]. SINO syndrome was first described in 3 children with three different de novo variants of the *KIDINS220* gene in 2016 in a study with 10 patients. Zhao et al. reported a family with a heterozygous *KIDINS220* mutation, who presented with spastic paraplegia but no signs of intellectual disability, nystagmus, or obesity [32]. Case 8 had a de novo *KIDINS220* mutation and presented with spastic paraplegia and intellectual disability without nystagmus and obesity (Table 2). This case broadens the clinical spectrum of *KIDINS220* variant-related disorders to encompass HSP with intellectual disability. On the other hand, Al Hussein H S et al. described a case of pure HSP with a likely pathogenic *KIDINS220* variant recently [33]. So, *KIDINS20*-associated variants may cause different phenotypes in which HSP is the main finding. Moreover, this case was the first Turkish patient with a *KIDINS220* gene mutation reported in the literature.

The protein encoded by the 4-hydroxyphenylpyruvate dioxygenase-like (*HPDL*) gene is localized to mitochondria and shows widespread tissue expression, with the highest levels in the brain [5,34]. The 4-hydroxyphenylpyruvate dioxygenase-like protein has been described to play a role in complex II activity and CoQ10 biosynthesis [4]. *HPDL* homozygous mutations causing *SPG83* were first reported in three unrelated Middle Eastern patients in 2020 [35]. Further, biallelic variants in the *HPDL* gene have been shown to cause a mitochondrial disease associated with neurological manifestations, as a syndrome varying from juvenile-onset pure HSP to infantile-onset spastic tetraplegia associated with global developmental delays [5,34]. As a result, biallelic variants in *HPDL* cause pure and complicated HSP. Case 9 was a 12-year-old girl with pure HSP associated with homozygous pathogenic mutation in the *HPDL* gene (Table 1). Shi G et al. have reported that 4-hydroxymandelate (4-HMA)-dependent CoQ synthesis is important for brain development and, as such, indicated that treatment with 4-HMA or 4-hydroxybenzoate (4-HB) in infancy or childhood may improve outcomes in patients with *HPDL* variants [35]. We were unable to access 4-HMA or 4-HB, but started the patient on Coenzyme Q10, vitamins B1 and B2, and biotin [4,5].

Biallelic mutations in the *CYP7B1* gene account for an important step in the alternative pathway of bile acid synthesis, resulting in a very rare form of HSP called *SPG5*. *CYP7B1* deficiency leads to the accumulation of oxysterols, which may be key pathogenic factors in *SPG5* [4,36,37]. It can present as both pure and complicated forms, and the age at onset showed both inter- and intrafamilial variations, with a range of 4–63 years [36,37,38]. Case 10 presented with developmental delay, developmental dysplasia of the hip, loss of appetite, fatigue, and gait disturbance since birth. On examination, her height and weight were below the third percentile, and spasticity and hyperreflexia were found in the lower extremities. All metabolic screening and spinal and brain MRI results were normal, and a new in silico pathogenic mutation in the *CYP7B1* gene was detected (Table 2). Marelli C et al. reported that plasma 25-hydroxycholesterol (25-OHC) and 27-hydroxycholesterol (27-OHC) are robust diagnostic biomarkers of *SPG5*, and should be used as first-line investigations in any patient with unexplained spastic paraplegia. In addition, they also assessed the combination of atorvastatin and chenodeoxycholic acid for the treatment of *SPG5* patients in a phase II therapeutic trial [6]. Although we could not measure 25-OHC and 27-OHC levels, we started ursodeoxycholic acid (UDCA) at the recommendation of our pediatric metabolism department.

A limitation of the current study was the relatively small sample size. Furthermore, there are generally limited data on genetically confirmed hereditary spastic paraplegia. Therefore, the phenotype–genotype correlations presented in this study might be helpful for the clinical and differential diagnosis of HSP, prediction of the disease course, monitoring symptoms, identifying targeted therapies, and providing genetic counseling. The highly variable duration of follow-up (from 8 months to 12 years) should be viewed as a further limitation regarding prediction of the subsequent clinical course in young patients.

## 5. Conclusions

In this paper, we report the first possible case of HSP associated with the heterozygous *SPG11* mutation in the literature. Although the *SPG11* mutation is usually associated with complicated HSP, this patient had a pure form of HSP. A possible heterozygous mutation with *GBA2*-associated HSP was also reported for the first time, which expanded the inheritance patterns. We also reported a novel homozygous *C19orf12* mutation associated with iron accumulation in the brain, which broadens the genetic variants and clinical findings. We determined the first Turkish patients with *CPT1C* and *TFG* gene mutation-related pure HSP. In addition, a *TFG* gene mutation was the second novel mutation related to the pure form of HSP identified for the first time in the literature. Case 8 was the first Turkish patient with the *KIDINS220* gene, thus broadening the clinical spectrum of *KIDINS220* variation-related disorders to encompass HSP with intellectual disability without nystagmus and obesity. Moreover, we suggested that HSP with the *KIDINS220* mutation might also be redefined based on the genetic locus classification via SPG numbering. A novel variant in the *CYP7B1* gene was detected in case 10, further expanding the genetic variants and clinical findings regarding *SPG5*.

## Figures and Tables

**Table 1:** Demographic and clinical findings of patients with HSP.

Patient No/ Sex	Clinical Follow-Up Ages					Family Medical History/ Consanguinity	Physical and Neurological Findings			Diagnosis	
	Age (y/m)	Presentation Age	Time of Progression of Symptoms	Follow Up Time	Age at Time of Diagnosis		Presentation Symptoms	Early Motor Development	Neurologic Findings	Initial	Final
1/F	7 y 6 m	2y	5 y	5 y 6 m	6 y 1 m	No/No	Gait disturbance and urine, stool incontinence	Able to walk at the age of 2	Spastic gait, brisk reflexes	HSP	SPG 11 P-HSP
2/M	4 y 7 m	6 m	2 y 3 m	4 y 1 m	2 y	No/Yes	Motor delay	No gain of ability to walk	Spastic gait, brisk reflexes, intellectual disability	DD	SPG46 C-HSP
3/F	9 y 10 m	1y	Stable disease	8 y 10 m	7 y	Yes/No	Gait disturbance	Able to walk at the age of 18 m	Spastic gait and brisk reflexes	HSP	SPG30 P-HSP
4/F	16 y 11 m	7y	7 y	9 y 11 m	14 y 2 m	Yes/Yes	Generalized weakness, falling frequently, and urine incontinence	No delayed motor milestones	Spastic gait, brisk reflexes, ataxia, ID, weakness in lower limbs	ID	SPG43 C-HSP
5/M	5 y 6 m	2 y 6 m	2 y 6 m	3 y	3 y	Yes/No	Gait disturbance and pain in lower limbs	No delayed motor milestones	Brisk reflexes, lower limb weakness	Spinal cord injury	SPG73 P- HSP
6/F	5 y 8 m	2 y 6 m	2 y 6 m	3 y 2 m	4 y 1 m	Yes/Yes	Tiptoe walking	No delayed motor milestones	Lower limb weakness, brisk reflexes, spasticity	HSP	SPG57 P-HSP
7/F	7 y 2 m	1 y	Stable disease	2 y 8 m	7 y	Yes/No	Delayed gait, Falling frequently	Able to walk at the age of 4, 5	Lower limb weakness, spasticity, Delayed speech, ID	CP	SPG4 P-HSP
8/F	6 y 3 m	Birth	Stable disease	4 y 3 m	5 y 5 m	Yes/No	Seizures, gait disturbance	No gain of ability to walk	Mild dysmorphic features, Spastic gait, brisk reflexes	Epilepsy, ID	SİNO syndrome C-HSP
9/F	12 y 9 m	12 y	12 y	8 m	12 y 1 m	Yes/No	Gait disturbance, falling frequently	No delayed motor milestones	Lower limb weakness, brisk reflexes, spasticity	HSP	SPG83 P-HSP
10/F	12 y 3 m	1 y 6 m	10 y	1 y 4 m	12 y 3 m	Yes/No	Gait disturbance, ID	Able to walk at the age of 2	Failure to thrive, mild dysmorphic features, lower limb weakness, spasticity	MD	SPG5 C-HSP

Abbreviation: HSP, hereditary spastic paraplegia; DD, Developmental delay; P, Pure; C, Complicated; ID, intellectual disability; CP, Cerebral palsy; MD, metabolic disease; y, year; F, female; M, male; m, month.

**Table 2:** Laboratory and genetic findings of patients with HSP.

Patient No	Neurologic Diagnostic Tests			Neuroimaging Tests		Genetic Findings	Treatment
	Metabolic Screening Tests	ENMG	EEG	Brain MRG	Spinal MRG		
1	-	-	-	Cerebellar hypoplasia, vermis dysplasia and corpus callosum (CC) hypoplasia	Normal	SPG11 c.6730C>T heterozygote (p.L2244F)	Physical therapy
2	Normal	Normal	Centrotemporal spike and waves on the left side with normal background	CC hypoplasia	Normal	GBA2 NM_020944.3 c.1688-2A>C heterozygote	Physical therapy, baclofen, diazepam, orthopedic braces, levetiracetam
3	Normal	-	Normal	Vermis and CC hypoplasia	Normal	KIF1A c.773C>T heterozygote (p.V391M)	Physical therapy, baclofen
4	Normal	Normal	Normal	Bilateral globus pallidus T2 hypointensity and iron accumulation	-	C19orf12c.385C>T (p.Q1239*)(p.Gln129Ter) Homozygote	Physical therapy, carbidopa/levodopa, baclofen,
5	-	-	-	Normal	Normal	CPT1C c.109C>T (p.R37C) heterozygote	Physical therapy
6	Normal	-	-	Normal	Normal	TFG NM_001195478.1 c.269-8_269-4dup Homozygote TFG NM_001195478.1 c.288_297 delCCTTGAATCAinsTGACTTG Homozygote	Physical therapy, baclofen, orthopedic braces
7	Normal	Normal	Normal	Normal	Normal	SPAST NM_014946.4 c.1496G>A heterozygote p.R499H	Physical therapy, baclofen orthopedic braces
8	Normal	-	Multifocal spike and waves pattern	Vermian, CC and brainstem hypoplasia	Normal	KIDINS220 NM_020738.4 c.4388C>A heterozygote p.S1463*	Physical therapy, clonazepam, sodium valproate
9	Normal	Normal	Normal	Normal	Normal	HPDL NM_032756.4 c.149G>A (p.Gly50Asp) Homozygote	Physical therapy, carbidopa/levodopa, baclofen, CoQ10, vitamin B1, B2 and biotin
10	Normal	Normal	Normal	Normal	Normal	CYP7B1 NM_004820.5 c.1346G>A Homozygotep.C449y	Physical therapy, ursodeoxycholic acid (UDCA), orthopedic braces

Abbreviations: HSP, hereditary spastic paraplegia; MRI, magnetic resonance imaging; CC, corpus callosum; ENMG, Electroneuromyography; EEG, Electroencephalography.

## Data Availability

Data is contained within the article.

## Abbreviations

HSP, hereditary spastic paraplegia; DD, developmental delay; ID, intellectual disability; CP, cerebral palsy; AD, autosomal dominant; AR, autosomal recessive; EEG, electroencephalography; ENMG, electroneuromyography; MRI, magnetic resonance imaging; SINO syndrome, spastic paraplegia, intellectual disability, nystagmus, obesity; HPDL, hydroxyphenylpyruvate dioxygenase-like.
